# Paediatric anti-neutrophil cytoplasmic antibody (ANCA)-associated vasculitis: an update on renal management

**DOI:** 10.1007/s00467-016-3559-2

**Published:** 2017-01-06

**Authors:** Lucy A Plumb, Louise Oni, Stephen D Marks, Kjell Tullus

**Affiliations:** 1 Department of Paediatric Nephrology, Great Ormond Street Hospital for Children NHS Foundation Trust, London, UK; 2 Department of Women’s and Children’s Health, Institute of Translational Medicine, Alder Hey Children’s Hospital, Eaton Road, Liverpool, L12 2AP UK

**Keywords:** ANCA vasculitis, Management, Glomerulonephritis, Paediatric, Diagnosis

## Abstract

The anti-neutrophil cytoplasmic antibody (ANCA)-associated vasculitides (AAV) are a group of disorders characterized by necrotizing inflammation of the small to medium vessels in association with autoantibodies against the cytoplasmic region of the neutrophil. Included in this definition are granulomatosis with polyangiitis (GPA, formerly known as Wegener’s granulomatosis), microscopic polyangiitis (MPA) and eosinophilic granulomatosis with polyangiitis (formerly known as Churg–Strauss syndrome). AAV are chronic, often relapsing diseases that can be organ or life threatening. Despite immunosuppression, the morbidity and mortality remain high. Renal involvement contributes significantly to the morbidity with high numbers of patients progressing to end-stage kidney disease. Current therapies have enabled improvements in renal function in the short term, but evidence for long-term protection is lacking. In MPA, renal involvement is common at presentation (90%) and often follows a more severe course than that seen in paediatric GPA. Renal biopsy remains the ‘gold standard’ in diagnosing ANCA-associated glomerulonephritis. While GPA and MPA are considered separate entities, the two are managed identically. Current treatment regimens are extrapolated from adult studies, although it is encouraging to see recruitment of paediatric patients to recent vasculitis trials. Traditionally more severe disease has been managed with the ‘gold standard’ treatment of glucocorticoids and cyclophosphamide, with remission rates achieved of between 70 and 100%. Other agents employed in remission induction include anti-tumor necrosis factor-alpha therapy and mycophenolate mofetil. Recently, however, increasing consideration is being given to rituximab as a therapy for children in severe or relapsing disease, particularly for those at risk for glucocorticoid or cyclophosphamide toxicity. Removal of circulating ANCA through plasma exchange is a short-term measure reserved for severe or refractory disease. Maintenance therapy usually involves azathioprine. The aim of this article is to provide a comprehensive review of paediatric AAV, with a focus on renal manifestations, and to highlight the recent advances made in therapeutic management.

## Introduction

The anti-neutrophil cytoplasmic antibody (ANCA)-associated vasculitides (AAV) are a group of disorders characterized by necrotizing inflammation of the small to medium vessels in association with autoantibodies against the cytoplasmic region of the neutrophil, namely proteinase 3 (PR3) and myeloperoxidase (MPO). Included in this definition are granulomatosis with polyangiitis (GPA, formerly known as Wegener’s granulomatosis), microscopic polyangiitis (MPA) and eosinophilic granulomatosis with polyangiitis (EGPA; formerly known as Churg–Strauss syndrome). AAV is a chronic, often relapsing disease that can be organ or life threatening [1, 2]. While modern immunosuppressive regimens have dramatically improved the prognosis for AAV, the associated disease and therapy-related morbidity and mortality remain high [3]. Renal involvement contributes significantly to the morbidity seen in paediatric AAV, with high numbers of patients progressing to end-stage kidney disease (ESKD). Current therapies have enabled improvements in renal function in the short term, but evidence for long-term protection is currently lacking. The disease burden associated with AAV is particularly important in children as they acquire educational achievements and strive to attain satisfactory growth, development and fertility, with the additional burden that they are likely to live with the disease and associated therapies for longer than their adult counterparts. The aim of this article is to provide a comprehensive review of paediatric AAV with a focus on its renal manifestations, as well as to highlight the recent advances made in therapeutic management.

## Diagnosis and classification

In 2005, the vasculitis working group of the Paediatric Rheumatology European Society (PRES), supported by the European League Against Rheumatism (EULAR) proposed new paediatric-specific classification criteria for GPA [4], which were validated in 2008 [5]. Major differences between EULAR/Paediatric Rheumatology INternational Trials Organisation (PRINTO)/PRES paediatric and the 1990 American College of Rheumatology (ACR) criteria were the addition of computed tomography imaging for pulmonary complications and more specific features for respiratory involvement [5]. The criteria were unable to differentiate MPA and EGPA from GPA due to the rarity of these subtypes, and as such no formal criteria for children are available. Current recommendations from the European Medicines Agency (EMA) to differentiate MPA from GPA are therefore based on an algorithm by Watts et al. [6], which has been validated in paediatric GPA and MPA cases [7].

In 2012, the nomenclature was updated: Wegener’s granulomatosis was renamed granulomatosis with polyangiitis, and Churg–Strauss syndrome became eosinophilic granulomatosis with polyangiitis [8]. Given the lack of validated criteria for AAV, the ACR criteria and Chapel Hill Consensus Conference definitions are used as such [9].

## Epidemiology

Although increasingly recognized, AAV remain rare. Due to changes in classification criteria, the true incidence is unclear. Primary systemic vasculitis (PSV) in UK children has an incidence of 10.6 cases per 100,000 population [10]. AAV are very rare; the estimated incidence of GPA in Europe is less than 1 per 2 million population per year [11], with a Canadian study suggesting a higher incidence at 6.39 per million population [12]. The incidence of MPA and EGPA in children is unknown [3] (Table 1).

**Table 1:** The incidence of anti-neutrophil cytoplasmic antibody-associated vasculitides in adult and childhood populations according to the literature

Reference	Country of study	Study period	Study population	Classification criteria	Incidence per 100,000 population		
					GPA	MPA	EGPA
Adult cohort data							
Watts et al. [12]	UK	1988–1997	All ages	CHCC/ACR	8.7–10.3	6.8–8.9	1.5–3.7
Watts et al. [13]	UK	1990–2005	All ages	Clinical diagnosis	8.4	-	-
Koldingsnes et al. [14]	Norway	1984–1998	Age >15 years	ACR	6.0–14.4	-	-
Gonzales-Gay et al. [15]	Spain	1998–2001	Age >15 years	CHCC	2.95	7.91	1.31
Reinhold-Keller et al. [16]	Germany	1998–2002	All ages	CHCC	6–12	2–3	0–2
Ormerod et al. [17]	Australia	1995–2004	Age >15 years	ACR/CHCC	8.4–8.8	2.3–5	2.2–2.3
Paediatric cohort data							
Gardner-Medwin et al. [18]	UK	1996–1999	Age <17 years	ACR	Primary systemic vasculitis incidence 10.6 per 100,000 patients (including ANCA-vasculitis)		
Grisaru et al. [19]	Canada	1994–2009	Age <18 years	ACR or EULAR/PRES	6.39	-	-

GPA, Granulomatosis with polyangiitis; MPA, microscopic polyangiitis; EGPA, eosinophilic granulomatosis with polyangiitis; CHCC, Chapel Hill Consensus conference; ACR, American College of Rheumatology; PRES, Paediatric Rheumatology European Society; EULAR European League Against Rheumatism; ANCA, anti-neutrophil cytoplasmic antibody

In adult-onset GPA, males are slightly more at risk than females [13]. The converse is seen in childhood disease, where GPA has a female predominance and tends to present around adolescence [1, 20]. Childhood MPA also peaks in early adolescence (12 years, range 7–17 years) [14, 15]. The geographical distribution of GPA has been shown to increase with increasing latitude, in both northern and southern hemispheres [16]. Ethnicity has also been associated with an increased risk: in a multi-ethnic population from France, GPA, MPA and EGPA were twofold more prevalent in subjects of European descent than in non-Europeans [17].

## Aetiology and pathogenesis

While several hypotheses have been posed, the exact pathogenesis of AAV remains unclear. Both PR3-ANCA and MPO-ANCA are strongly associated with AAV although they may correlate with separate phenotypes: PR3-ANCA is associated with a granulomatous vasculitis whereas MPO-ANCA correlates with a necrotizing small-vessel vasculitis. Granulomata are not a feature of MPO-ANCA-associated vasculitis. There is increasing evidence that ANCA may play a primary role in the development of necrotizing small-vessel vasculitis [18]. In vitro studies have demonstrated that ANCA can stimulate neutrophils to produce reactive oxygen species and lytic enzymes [21]. Before this can occur, pro-inflammatory cytokines [tumour necrosis factor-alpha (TNF-α), interleukin (IL) 1 and IL 18] must prime the neutrophils, leading to the upregulation of neutrophil adhesion molecules (CD11b) and translocation of PR3/MPO antigens to the neutrophil surface membrane. Subsequent interaction between ANCA and the relevant ANCA-antigen activates the neutrophil, causing increased vessel wall adherence and transmigration. The ensuing ANCA-mediated activation results in neutrophil degranulation and release of reactive oxygen species, resulting in vasculitis [19]. In vivo experimental studies have since confirmed a role for MPO-ANCA in the development of necrotizing vasculitis [18].

Observations that infectious episodes may trigger relapses of AAV have led to the hypothesis that microbial factors may play a role in disease pathogenesis. This possibility is supported by placebo-controlled adult studies that demonstrate a significant reduction in relapses for patients treated with co-trimoxazole [22, 23].

Following reports of familial associations, evidence for genetic susceptibility is increasing. A genome-wide association study performed in 1233 UK patients with AAV and replicated in 1454 Northern European patients has demonstrated genetic associations with AAV and revealed that GPA and MPA are two genetically distinct conditions [24]. The strongest genetic associations were found to correlate with the antigenic specificity of ANCA rather than the clinical phenotype, with anti-PR3-ANCA correlating with *HLA-DP* and the α-antitrypsin gene (*SERPINA1*, a serine proteinase inhibitor for which PR3 is one of the substrates) and proteinase 3 (*PRTN3*) and anti-MPO-ANCA associated with *HLA-DQ* [24].

## Clinical features

The clinical features of disease in children and adults with GPA and MPA, respectively, in Table 2.

**Table 2:** The clinical features of disease in children and adults with granulomatosis with polyangiitis and microscopic polyangiitis, respectively

Granulomatosis with polyangiitis						
Disease feature	Paediatric data					Adult data
	Rottem et al. [25] (*n* = 23)	Stegmayr et al. [21] (*n* = 10)	Belostotsky et al. [2] (*n* = 17)	Akikusa et al. [1] (*n* = 25)	Cabral et al. [24] (*n* = 65)	Fauci et al. [26] (*n* = 18)
Criteria used for diagnosis	ACR 1990	Triad of RPCGN, raised ANCA and upper/lower respiratory disease	ACR 1990	ACR 1990	ACR 1990	NR
Median age at onset (years)	15.4	16.5	6	14.5	14.2	43.6
Male:female	1:2.1	1:1	1:3.25	1:4	NR	1:0.6
Median duration of symptoms prior to admission, in months (range)	NR	1 (0–2.5)	NR	2 (0.3–12)	2.7 (0–49)	NR
Constitutional	NR	10 (100)	NR	24 (96)	58 (89.2)	14 (78)
Pulmonary	5 (22)	9 (90)	14 (82.4)	20 (80)	52 (80)	18 (100)
Upper airway	20 (87)	10 (100)	17 (100)	21 (84)	52 (80)	18 (100)
Renal	2 (9)	10 (100)	4 (23.5)	22 (88)	49 (75.4)	15 (83)
Requiring RRT	0 (0)	NR	1 (5.8)	5 (20)	NR	NR
Skin	NR	4 (40)	9 (53)	8 (32)	23 (35.4)	8 (44)
Neurological	NR	2 (20)	2 (12)	2 (8)	16 (24.6)	4 (22)
Gastrointestinal	NR	NR	NR	3 (12)	27 (41.5)	NR
Joints	NR	5 (50)	NR	NR	37 (56.9)	10 (56)
Eyes	3 (13)	0 (0)	9 (53)	13 (52)	24 (36.9)	7 (39)
Cardiovascular	NR	NR	NR	3 (12)	0 (0)	5 (28)
PR3-ANCA positive	NR	8 (80)	10 (59)	13 (86.7)	40 (93)	NR
MPO-ANCA positive	NR	1 (10)	NR	2 (13.3)	0 (0)	NR
Microscopic polyangiitis						
Disease feature	Paediatric data				Adult data	
	Bakkaloglu et al. [27] (*n* = 10)	Hattori et al. [28] (*n* = 21)	Peco-Antic et al. [29] (*n* = 7)	Siomou et al. [30] (*n* = 6)	Nachman et al. [31] (*n* = 69)	
Criteria used for diagnosis	NR	Derived from 1994 CHCC classification criteria	Triad of clinical features of small-vessel vasculitis, biopsy proven pauci-immune necrotizing glomerulonephritis and raised MPO-ANCA titres	Derived from 1994 CHCC and EULAE/PRES classification criteria	Derived from 1994 CHCC classification criteria	
Median age at onset (years)	9.5	11.9	12	11.6	57.6	
Male:female	1:1.5	1:6.8	0.16:1	0:6	1:0.83^a,^ ^ b^	
Median duration of symptoms prior to admission, in months (range)	NR	NR	6.7 (3–15)	NR (1–12)	NR	
Constitutional	10 (100)	18	7 (100)	NR	NR	
Pulmonary	3 (30)	13	4 (57)	3 (50)	38	
Upper airway	NR	2	NR	1 (16.7)	14	
Renal	7 (70)	21	7 (100)	6 (100)	69	
Requiring RRT	0 (0)	7 ()*	2 (29)	1 (16.7)	6 (5.6)^a^	
Skin	7 (70)	8	7 (100)	NR	13	
Neurological	2 (20)	1	6 (86)	1 (16.7)	9	
Gastrointestinal	2 (20)	7	4 (57)	NR	6	
Joints	6 (60)	2	NR	NR	10	
Eyes	NR	2	NR	1 (16.7)	2	
Cardiovascular	NR	NR	NR	NR	NR	
PR3-ANCA positive	0 (0)	3 (9.7)*	0 (0)	0 (0)	36 (37.1)^a,^ ^ b^	
MPO-ANCA positive	9 (90)	31 (90.3)*	7 (100)	5 (83.3)	61 (62.9)^a,^ ^ b^	

Values in table are given as the number of patients with the percentage of the respective cohort given in parenthesis, unless indicated otherwise

ACR 1990, ACR 1990 criteria [5]; RPCGN, rapidly progressing crescentric glomerulonephritis; NR, not recorded; RRT renal replacement therapy; PR3, proteinase-3; MPO, myeloperoxidase; NCGN, necrotizing and crescentic glomerulonephritis

^a^Included in the calculation were patients diagnosed with MPA and NCGN (or ‘renal-limited’ vasculitis) (* n* = 107)

^b^Calculation includes only those in study who received treatment (*n* = 97/107 total MPA/NCGN)

### Granulomatosis with polyangiitis

The differences between paediatric GPA and MPA presentation and adult data are shown in Table 2 [27, 29]. In 2007 a collaboration between U.S. and Canadian centres led to the establishment of the ARChiVe (A Registry for Childhood Vasculitis: e-entry) vasculitis registry. This multi-centre group have since described the largest cohorts of paediatric GPA to date [20, 32].

Characteristic features of both adult and childhood GPA include necrotizing granulomata of the upper and lower respiratory tract, necrotizing vasculitis and glomerulonephritis (GN). Childhood GPA differs from adult GPA in several key features [5]. Childhood disease is frequently heralded by the presence of constitutional symptoms such as fever, anorexia and weight loss [1]. Multi-organ or generalized disease is common, consisting predominantly of ear, nose and throat (80%), pulmonary (80%) and renal (75.4%) involvement, respectively. Less frequently involved are the musculoskeletal (57%), gastrointestinal (42%), eyes (37%), skin (35%) and nervous systems (25%) [20].

Upper airway involvement in children is consistently reported at presentation, with an incidence of 70–100% [20, 32], commonly manifesting as recurrent epistaxis or sinusitis. Oral ulceration is also reported although without evidence of granulomata on biopsy [2]. Chronic inflammation can result in nasal septum perforation, saddle-nose deformity, chronic sinusitis and conductive hearing loss [1]. Respiratory symptoms described in the ACR-defined ARChiVe cohort and other studies include dyspnoea (59%) and chronic cough (52%), hoarseness (12%), stridor (29%) and haemoptysis (18%), [2, 20].

In those children whose radiographs show changes, findings include diffuse pulmonary infiltrates, nodules, cavitating lesions and granulomata without cavitation. Pulmonary nodules and haemorrhage are reported with a similar incidence [1, 20]. Severe pulmonary haemorrhage requiring mechanical ventilation can occur in 16–20% of patients at presentation. In comparison with adult disease, subglottic stenosis is a highly specific feature of paediatric GPA, reportedly affecting up to 50% of paediatric patients [33], and can present independently of systemic disease [34]. As such, it has been included in new classification criteria [5].

Renal manifestations of childhood GPA contribute to significant morbidity. Presentation can vary from urinary sediment abnormalities or mild renal dysfunction to acute kidney injury requiring renal replacement therapy (RRT) [1, 20, 32]. A diagnosis of glomerulonephritis based on biopsy findings is common. In a single-centre review of biopsy-proven ANCA-associated glomerulonephritis, significant acute kidney injury [glomerular filtration rate (GFR) <60 ml/min/1.73 m^2^) and/or nephrotic-range proteinuria at presentation was found in 57% (4 of 7) patients with childhood GPA [35]. While numbers were limited, oliguria, markedly reduced GFR, nephrotic-range proteinuria and chronic glomerular lesions were associated with poorer outcomes [35].

Arthralgia and myalgia are reported in up to 64% of patients at presentation and are deemed to be early markers for disease flare. Arthritis is less common (20–32%) and seen in the context of active disease [1]. Ocular involvement has been reported in up to 53% of paediatric patients. Inflammatory changes can occur in any compartment causing proptosis [36], conjunctivitis, episcleritis and uveitis. Venous thrombosis is an uncommon finding, having been reported in 12% of paediatric patients [1]. Cutaneous involvement affects up to 53% of patients at presentation and includes palpable purpura, which is often mistaken for immunoglobulin A (IgA) vasculitis (the new term for Henoch–Schönlein purpura), petechiae and nodules. Nervous system involvement is uncommon although symptoms can range from headache and dizziness to acute mononeuritis. Cerebellar involvement, seizures and upper motor neurone signs have all been described in children with GPA [1, 2].

### Microscopic polyangiitis

Microscopic polyangiitis is a rare disease in childhood and is characterized by a multi-system pauci-immune necrotizing small-vessel vasculitis without granulomatous inflammation. Prior to ARChiVe, evidence of paediatric presentation and disease course in MPA was limited [14, 15]. Recently, however, the ARChiVe Investigators Network described the largest paediatric MPA cohort to date (48 patients) using EMA criteria [32].

As with GPA, a female predominance is seen in childhood (as high as 6:1 [14]). Patients are significantly younger than those with GPA (11 vs. 14 years) [32]. Onset is insidious and associated with constitutional symptoms in almost all patients [15, 32]. Purpuric rash at presentation is common although other skin lesions include necrotizing vasculitis and lesions mimicking pyoderma gangrenosum [14]. The incidence of central nervous system involvement has varied between cohorts, ranging from 21 to 86%. In the cohort study by the ARChiVe Investigators Network symptoms included seizures, optic neuritis and peripheral neuropathy. Pulmonary symptoms including cough, haemoptysis and dyspnea were present in 44% of cases, although symptoms were less frequent and severe than in GPA patients. Using EMA criteria, gastrointestinal symptoms including chronic nausea and non-specific abdominal pain were also seen in the majority of patients (58%) [32].

Renal involvement is common at presentation (75–90%) [14] and often follows a more severe course than that seen in paediatric GPA [32, 35]. In the ARChiVe Investigators Network study, three-quarters of patients described using EMA criteria demonstrated renal involvement, with manifestations including proteinuria, microscopic haematuria and renal dysfunction with moderate to severely elevated serum creatinine levels (>30% age-adjusted upper limit of normal) in 48% of affected patients. Hypertension was noted in one-third of cases, while RRT was required in one-quarter of patients at presentation. Delay in diagnosis and higher Birmingham vasculitis activity scores (BVAS) have been associated with poor renal outcome [14].

Bakkaloglu et al. reported a high incidence of acute kidney injury at presentation (6/10 patients), with one-half of the patients requiring RRT in association with pulmonary involvement and high MPO-ANCA titres [150–250 endotoxin units (EU)/ml]. All patients with pulmonary–renal involvement progressed to end-stage kidney disease (ESKD) within 1 to 5 years. Hypertension was present in 83% (5 of 6) MPA patients with renal involvement, with evidence of renal artery aneurysms in one patient [15].

### Renal limited vasculitis

Renal limited vasculitis (RLV) is a term used for renal histopathology [previously classified as pauci-immune, necrotizing crescentic (NCGN) or ANCA-associated glomerulonephritis (AAGN)] in the absence of vasculitis in other organs. Approximately 80% of patients with RLV will be positive for ANCA, predominantly MPO-ANCA, and as such may be cohorted with patients diagnosed as MPA [37]. In children, AAGN is the second commonest cause of rapidly progressive glomerulonephritis (RPGN) behind immune-mediated pathologies, such as post-infectious, IgA vasculitis and IgA nephropathy. In a single-centre study of RPGN, pauci-immune crescentic GN was observed in 31/73 patients aged between 1 and 20 years [37]. In a retrospective study for the Japanese Society of Paediatric Nephrology, Hattori et al. reported a mean age at presentation of 11.9 years (Table 2) and female preponderance (6.8:1). Of the ten patients described, seven were detected through national screening, while three presented with constitutional symptoms of malaise, anorexia, fever and weight loss. During a median follow-up period of 3.9 (range 1.3–4.9) years, no patients developed evidence of extra-renal disease [38].

### Eosinophilic granulomatosis with polyangiitis

Eosinophilic granulomatosis with polyangiitis is extremely uncommon in children and is thought to contribute 2% of all paediatric PSV. It is a necrotizing vasculitis of small- and medium-sized vessels that predominantly affects the upper airways and lungs. Extra-pulmonary disease including cutaneous, cardiac, neurological and gastrointestinal involvement is common. The presence of ANCA is less common in paediatric EGPA than in adult patients, with 0–25% cases testing positive. To date, 47 cases have been published within the paediatric literature; none of which had evidence of renal involvement [39]. We therefore aim to focus further discussion within this review on the management of GPA and MPA only.

## Renal histology in AAV

Renal biopsy remains the ‘gold standard’ in diagnosing AAGN. Classically, AAGN is characterized by a pauci-immune crescentic necrotizing glomerulonephritis on light microscopy. Histopathological features may vary, including acute lesions such as glomerular crescents or tubular intra-epithelial infiltrates to lesions indicative of chronic inflammation, i.e. glomerulosclerosis, interstitial fibrosis or tubular atrophy [26]. Electron microscopy features include subendothelial oedema, microthrombi and degranulation of neutrophils [31]. While a paucity of immune deposits is considered typical, there are a few cases of childhood AAV in which immune deposition has been seen on examination by electron microscopy [35]. It is speculated that their presence may somehow potentiate the effect of ANCA in the development of glomerulonephritis [25].

Glomerulonephritis in MPA patients tends to be associated with a greater degree of chronic lesions, whereas a greater proportion of normal glomeruli are seen in GPA. In a paediatric study of MPA, findings included focal segmental necrosis of glomeruli among those children with a shorter duration between symptom onset and diagnosis, while those with greater delay demonstrated circumferential fibrous and/or fibro-cellular crescents [15]. This finding is supported by other studies [35, 38]. Glomerulosclerosis is also more prominent in patients positive for MPO-ANCA, which suggests the pathogenesis of renal disease may differ between ANCA subtypes [26].

Adult studies have attempted to delineate the prognostic value of pathologic lesions on biopsy alongside patient demographics. Baseline GFR has been shown to correlate with renal function at 18 months of follow-up [40]. ANCA subtype was found not to be independently linked to renal prognosis. The percentage of normal glomeruli has been shown to strongly correlate with renal function [30] at baseline and follow-up [28]. In terms of acute lesions, the presence of cellular crescents is associated with improvements in renal function independent of baseline GFR, suggesting a potential reversibility of active disease [40]. Conversely, fibrous crescents correlate with RRT requirement at baseline and a poor renal outcome [28]. Chronic lesions including glomerular sclerosis and tubular atrophy, as well as degree of proteinuria at baseline relate to poor long-term outcomes [30, 41].

Collating this information, an international working group in 2010 proposed a new histopathologic classification for AAGN that features four distinct categories: focal, crescentic, mixed and sclerotic [42–44]. This classification system has since been validated in children with AAGN over a median follow-up period of 2.4 years, demonstrating a probability of an estimated GFR (eGFR) of >60 ml/min/1.73 m^2^ at 2 years as 100% for focal lesions, 56.5% for crescentic/mixed and 0% for sclerotic biopsy categories [45].

## Measurement of disease activity

Formal measurement of disease activity is essential to assess treatment efficacy in clinical practice and therapeutic trials. In adults, the BVAS and Vasculitis Activity Index are disease tools that have been validated in AAV [46, 47]. These tools have undergone a number of changes, including disease-specific modifications for GPA (formerly known as BVAS-WG).

Recently International collaborative working groups have emphasized the need to develop a robust paediatric-specific disease measurement tool. The development of the Paediatric Vasculitis Activity Scale has shown strong correlation with physician assessment, erythrocyte sedimentation rate and treatment decision, as well as high agreement between assessors [48].

## Investigations

Primary systemic vasculitis should be considered in any child presenting with a multi-system disease. Investigations which should be performed in children when there is a suspicion of AAV are listed in Table 3. It is important to exclude differential diagnoses such as infection or malignancy.

**Table 3:** Suggested investigations in suspected anti-neutrophil cytoplasmic antibody-associated vasculitis

First-line investigations	Second-line investigations
Haematology -Full blood count with blood film -Erythrocyte sedimentation rate -Coagulation profile	Biochemistry/Immunology -Basic lymphocyte subsets -CD19 count (pre-rituximab) -Hepatitis B&C, parvovirus B19, human immunodeficiency virus -Mantoux and/or quantiferon tuberculosis testing -Ebstein–Barr virus, cytomegalovirus, enterovirus, adenovirus, JC virus
Biochemistry -Urea & electrolytes -Liver function tests -Lactate dehydrogenase -Creatinine kinase -Thyroid function -Pancreatic function (amylase/lipase) -Urine albumin:creatinine ratio	Imaging -Computed tomography/magnetic resonance imaging (MRI) /X-ray sinuses and/or thorax -Renal dimercaptosuccinic acid scan -MRI/MR angiography head
Infectious disease screen -Blood culture -Urine microscopy and cell culture -Mycoplasma serology -Anti-streptolysin titre -Anti-DNase B	Neurological -Nerve conduction studies
Immunological tests -Anti-neutrophil cytoplasmic antibodies -Antinuclear antibodies -Anti-extractable nuclear antibodies -Rheumatoid factor -Anti-double stranded DNA antibodies -Anti-glomerular basement membrane antibodies -Coeliac screen -Complement (C) function: C3/4, CH100, Mannose binding lectin -Anti-cardiolipin antibodies -Lupus anticoagulant -Varicella status -Immunoglobulins (Ig): IgG, IgM, IgA, IgE -Serum angiotensin converting enzyme	Other -Histology: renal/lung biopsy -Ambulatory blood pressure monitoring -Formal glomerular filtration rate testing
Other -Chest X-ray -Electrocardiography -Echocardiography	

Immunological testing is important in the work-up of ANCA-associated and PSV. ANCA positivity by enzyme-linked immunosorbent assay or indirect immunofluorescence testing in the paediatric population has a sensitivity 93% and specificity of 90% [5]. GPA is frequently associated with elevated titres of ANCA directed at PR3 that lead to cytoplasmic staining of neutrophils (cANCA), whilst MPA is frequently associated with antibody directed against MPO that stains neutrophils in a perinuclear fashion (pANCA) [49].

Glomerulonephritis may present as haematuria and/or proteinuria on dipstick testing, or in a more pronounced fashion, with hypertension, oligo-anuria and/or nephrotic-range proteinuria. Early renal biopsy should be considered in patients with renal involvement.

## Management

Modern immunosuppression has increased the survival rates of patients with AAV. Treatment is based on intensive remission induction followed by maintenance therapy.

**Fig. 1:**
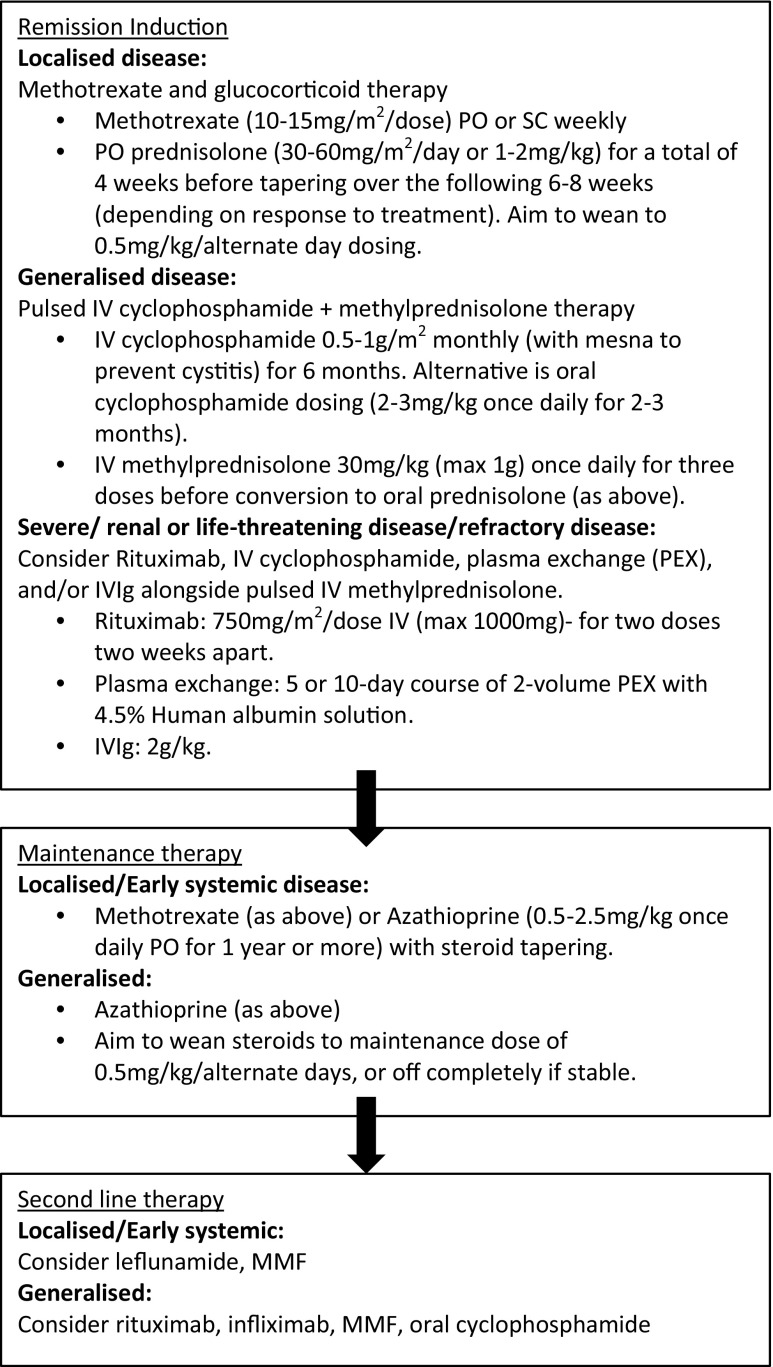
A treatment algorithm for anti-neutrophil cytoplasmic antibody-associated vasculitis according to the severity of the presenting disease and the treatment for both remission induction and maintenance of disease.* PO* Oral,* SC* subcutaneous,* IV* intravenous,* IVIg* intravenous immunoglobulin,* MMF* mycophenolate mofetil

At present, there are very little data to allow the development of a paediatric-specific AAV guideline. The SHARE project, a European initiative, aims to provide treatment recommendations for the care of children and young adults with vasculitis. These recommendations are currently awaited and will be based on surveys sent to PRINTO members of current clinical practice and systematic literature reviews [50]. Therefore, this review will provide information based on the authors’ experience and the evidence obtained from the limited number of randomized controlled trials (RCTs) performed in adults extrapolated to children (Fig. 1). While GPA and MPA are considered separate entities, the two are managed identically with regards to therapeutic intervention.

A recent study from the ARChiVE group demonstrated significant concordance between treatment intensity and the European Vasculitis Study Group (EUVAS) subgroupings in 125 paediatric patients [51]. Therefore, we used these disease categories to describe therapeutic options (Table 4).

**Table 4:** European Vasculitis Study Group definitions used in cases of anti-neutrophil cytoplasmic antibody-associated vasculitis based on the severity and extent of disease

Category	Description
Localized	Upper and/or lower respiratory symptoms without other system involvement or constitutional symptoms
Early systemic	Disease without threatened organ function or life-threatening disease (also known as ‘non-organ or life threatening’
Generalized	Renal, or other organ-threatening disease. Serum creatinine <500 μmol/l (or 5.6 mg/dl)
Severe	Renal or other organ failure, serum creatinine >500 μmol/l (5.6 mg/dl). Also known as ‘immediately life threatening’
Refractory	Disease unresponsive to glucocorticoids and cyclophosphamide

### Remission induction

The initial goal of therapy in AAV is to achieve remission, formally defined using disease activity tools. Traditionally severe disease including renal involvement has been managed with the ‘gold standard’ treatment of glucocorticoids and cyclophosphamide, with remission rates of between 70 and100% [43, 52–54]. Other agents employed in remission induction include TNF-α therapy and mycophenolate mofetil (MMF). Recently, however, increasing consideration is being given to rituximab as an equally effective therapy in severe or relapsing therapy, particularly for those patients at risk for glucocorticoid or cyclophosphamide toxicity.

Patients with severe or generalized disease have historically received large amounts of immunosuppression given their poor prognosis, including intravenous (IV) cyclophosphamide and methylprednisolone [52]. In such cases, adjuvant therapy in the form of plasma exchange has been shown to increase the renal survival in this cohort [53].

#### Cyclophosphamide

Cyclophosphamide is an alkylating agent that has long held a key role in the remission induction and maintenance of PSV and AAV. However, concerns do exist regarding cumulative toxicity, particularly in children. High cumulative doses can result in unacceptable adverse events, such as haemorrhagic cystitis (43%), infertility (57% of females), opportunistic infections (0.11 infections per patient-year) and a 2.4-fold increased risk of malignancy, among others [54].

Research has focused on minimizing cumulative doses, as well as determining equally effective alternatives. In AAV, a meta-analysis has confirmed that IV cyclophosphamide pulse therapy is as effective as cyclophosphamide administered as daily doses and results in a smaller cumulative dose with fewer adverse effects [55, 56].

#### Anti-TNF-α therapy

In AAV, it is thought that TNF-α is likely to play a role in neutrophil priming, resulting in the expression of endothelial adhesion molecules and ANCA antigens on the cell surface [18]. The Wegener’s granulomatosis etanercept trial (WGET) assessed the efficacy of etanercept, a TNF-α blocker, in remission induction and remission maintenance of GPA [57, 58]. A total of 108 patients were randomized to receive either etanercept or placebo in addition to standard therapy (cyclophosphamide and corticosteroids for severe disease or methotrexate and corticosteroids for limited disease). No difference in remission induction rates were seen between the two groups although an increase in solid cancers was noted in those patients receiving standard therapy combined with cyclophosphamide, suggesting a cumulative malignancy risk. Relapse rates during maintenance therapy were similar.

Interestingly, whilst inhibition of TNF-α via etanercept has shown no benefit in remission induction for either early systemic or generalized disease, anti-TNF-α antibodies, such as infliximab and adalimumab, do show promise. In an open-label prospective trial of 16 AAV adults with renal involvement, adjuvant infliximab therapy was noted to achieve a mean time-to-remission of 6.4 weeks when used alongside cyclophosphamide and enabled a significant reduction of steroid doses [59]. However, there have been no RCTs to confirm this finding to date. The results of a prospective open-label study of adjuvant adalimumab suggest that while remission rates are similar to those seen with standard therapy alone (78.5% by week 14), a significant steroid-sparing effect is present [60].

#### Mycophenolate mofetil

At present, there is limited evidence to support the use of MMF in induction regimens for AAV. Recently, the results of a non-inferiority trial were reported (‘MYCYC’; a randomized control trial of MMF vs. cyclophosphamide in remission induction in AAV) [61]. The study comprised 140 newly diagnosed patients (70 per arm), including paediatric patients. The primary outcome was remission, defined as absence of disease activity for longer than 4 weeks when adhering to the glucocorticoid regimen. The study was unable to demonstrate that MMF is non-inferior to cyclophosphamide when adherence is taken into account; however, the authors conclude that further evaluation of how glucocorticoid treatment affects remission induction is warranted [61].

#### Plasma exchange

Removal of circulating ANCA through plasma exchange is a short-term measure often reserved for severe or refractory disease. It should be used in conjunction with immunosuppression. The MEPEX trial consisted of 137 patients with severe renal vasculitis who were randomized to receive plasma exchange versus pulsed methylprednisolone [44]. The results demonstrated a 50% relative reduction in the need for RRT at 12 months in those who received plasma exchange, with no difference in the frequency of adverse effects. The authors of a meta-analysis of nine studies concluded that while renal survival with plasma exchange is encouraging (overall relative risk of renal disease following adjuvant therapy 0.64, 95% confidence interval 0.47–0.88,* p* = 0.006), there is insufficient evidence to suggest whether it improves overall survival [53, 62], highlighting the need for larger studies. At the present time there is little evidence of the effect of plasma exchange on extra-renal manifestations [63]. An international open label study is currently recruiting to address these questions [64]. With the aim of recruiting 700 patients, ‘PEXIVAS’ is a randomized trial with the intent to compare adjuvant plasma exchange and two oral glucocorticoid regimens in patients undergoing remission induction treatment, including adolescents (aged >15 years). The primary outcome measure is mortality from any cause and ESKD [64].

### Maintenance therapy

Studies have confirmed findings that cyclophosphamide can safely be substituted for maintenance azathioprine, thereby avoiding large cumulative doses and their associated toxicity [43]. Other options for remission maintenance in milder disease without renal involvement include methotrexate and leflunamide. MMF may be considered for patients in whom azathioprine is poorly tolerated, although it appears less efficacious [65].

#### Azathioprine

In terms of the maintenance of disease control, the CYCAZAREM trial (randomized trial of Cyclophosphamide versus Azathioprine during Remission in ANCA-positive systemic vasculitis) demonstrated no significant differences in rates of relapse between patient groups at 18 months [15.5 vs. 13.7% in cyclophosphamide and azathioprine (AZA) groups, respectively] [43]. Subsequent retrospective studies with longer follow-up periods demonstrated a slightly higher relapse rate in patients treated with azathioprine (42.3 vs. 57.4%) at 5 years [66]. Many of the relapses reported however occurred following discontinuation of therapy and were more frequent in patients positive for PR3-ANCA when starting therapy.

#### Mycophenolate mofetil

The IMPROVE study (International MMF Protocol to Reduce Outbreaks of Vasculitides), an open-label, multi-center RCT, was designed to assess whether MMF reduced the risk of relapse compared with azathioprine for AAV patients in remission [65]. The results demonstrated an inferior effect to that of AZA in sustaining disease-free survival (55.3 vs. 37.5%). No differences in the number of adverse effects were reported, but in view of these results, AZA is generally preferred over MMF for remission maintenance. As an alternative, and in those with milder disease (including adequate renal function), methotrexate may be safely used for maintenance of remission in this cohort [63]. Leflunamide is reported to be more efficacious than methotrexate, but it is associated with greater adverse effects [67].

### Management of refractory disease

Similar to severe disease, progressive, unresponsive disease in AAV is associated with significant mortality that is both disease- and therapy-related. In these circumstances, further courses of induction treatments should be considered, such as IV cyclophosphamide, methylprednisolone, infliximab, and plasma exchange. Biologic agents play a significant role in these patients.

#### Rituximab

There is increasing evidence that rituximab has a short- and medium-term benefit in both severe and refractory disease. For children who are at particular risk of toxicity from both cyclophosphamide and corticosteroids, rituximab is likely to provide an alternative, equally efficacious means by which disease activity can be managed. In AAV, chronic T-cell activation is thought to promote maturation of auto-reactive B cells, which in turn leads to the production of ANCA. Rituximab, a chimeric monoclonal anti-CD20 antibody, causes B cell depletion and has been evaluated in two RCTs for remission induction—RAVE and RITUXVAS [68, 69]. A further evaluation of its use in remission maintenance, the MAINRITSAN study, has also been published with positive results [70].

The RAVE trial (Rituximab in ANCA-associated Vasculitis) was a multi-center RCT designed to assess the use of rituximab versus cyclophosphamide in remission induction [69]. New patients or those with relapsing disease were randomized to receive either rituximab (375 mg/m^2^ IV weekly for 4 weeks) or oral cyclophosphamide (2 mg/kg/day). Patients with severe renal disease were excluded. Patients in the cyclophosphamide arm who achieved remission between 3 and 6 months were eligible to switch to AZA for the remainder of the study whilst the rituximab group received placebo. A total of 197 AAV patients were recruited. Rituximab was found to be non-inferior when compared with cyclophosphamide for achieving disease remission by 6 months. In patients with relapsing disease, rituximab was superior. No difference was seen with regards to adverse effects or steroid exposure.

The RITUXVAS trial compared rituximab (375 mg/m^2^ IV weekly for 4 weeks administered with 2 × 15 mg/kg IV cyclophosphamide) to the ‘gold standard’ IV cyclophosphamide regimen (6–10 doses at 15 mg/kg) for new AAV patients and included those with renal involvement [68]. The results demonstrated that rituximab is as effective as cyclophosphamide in inducing remission, with both achieving high remission rates (76% in rituximab arm vs. 82% in cyclophosphamide arm). Similar numbers of adverse effects were reported (18% mortality in both treatment arms). Further data from the RITUXVAS group at 2 years of follow-up has revealed no differences between rituximab and control with regards to a composite outcome of death, ESKD or relapse. While no relapses were demonstrated in B-cell-depleted patients, B-cell return alone was not felt to be a sufficient marker for potential relapse: only 30% of patients with B-cell return had a confirmed clinical relapse. No differences in patients achieving ANCA negativity were noted (88% in rituximab vs. 73% in cyclophosphamide group) [71].

Subsequent prospective data from the RAVE group demonstrates that there is no difference in remission rates when rituximab was given to patients from both treatment arms for relapse. This result suggests that the effect of rituximab is not dependent on the initial remission-induction agent used. Again, ANCA and B-cell counts were not predictive of relapse [72].

With respect to remission maintenance, rituximab has been compared in a non-blinded RCT with AZA in the MAINRTISAN study (Maintenance of Remission using Rituximab in Systemic ANCA-associated vasculitis) [70]. Eligible patients included those with biopsy-proven ANCA-positive GPA, MPA or renal-limited AAV. Following the induction of remission, patients were randomized to receive either fixed doses of rituximab 500 mg at days 0 and 14 and months 6, 12 and 18 after first infusion, or control AZA at 2 mg/kg daily for 12 months, followed by 1.5 mg/kg daily for a further 6 months. The primary outcome was percentage of patients who experienced a major relapse (BVAS score >0 and involvement of one major organ) at month 28. A total of 115 patients underwent randomization, with each group having similar baseline characteristics and proportions of relapsing disease. There were fewer major relapses in the rituximab group than in the control group (hazard ratio for relapse 6.61, 95% confidence interval 1.56–27.96,* p* = 0.002), with similar severe adverse effects noted in both groups.

An international open-label RCT comparing rituximab and AZA as maintenance therapy in relapsing AAV is currently recruiting (the ‘RITZAREM’ study). Remission induction will be achieved with rituximab (4 × 375 mg/m^2^) followed by randomization to fixed-interval repeat dosing or AZA for 24 months. It is anticipated that 160 patients will be recruited for a follow-up study period of 4 years. Retrospective evidence suggests fixed-interval dosing confers a greater clinical benefit to patients without additional adverse effects, although randomized studies are required to confirm this finding [73].

In refractory disease, adjuvant therapy with infliximab or rituximab alongside conventional immunosuppression has been compared in a prospective RCT. Although the trial involved only a small number of patients, results suggested rituximab was more effective at obtaining and sustaining remission over a mean follow-up of 31 months [74].

Rituximab use has been described in 13 paediatric patients. In one study, the administration of rituximab resulted in significantly improved BVAS scores and enabled steroid weaning in four children with GPA [75]. Other case series report high remission rates for both GPA and MPA even when conventional treatments have failed. A phase IIa international multi-center open-label trial is currently ongoing to evaluate the safety and pharmacokinetics of rituximab in children with severe AAV (The ‘PEPRS’ study; NCT01750697). Newly diagnosed patients or those with relapsing disease who have not previously received rituximab will be eligible for inclusion. Recruitment commenced in 2013 and follow-up will continue for approximately 3.5 years.

Current expert consensus recommends the use of rituximab for remission induction in refractory disease, or where there are concerns regarding cumulative cyclophosphamide or corticosteroid toxicity, particularly in children [76, 77]. Recommendations also include repeat treatment where relapse occurs following a rituximab-induced remission.

#### IV Immunoglobulin

Intravenous immunoglobulin (total dose 2 g/kg) as adjuvant therapy was effective at producing a significant short-term improvement in disease activity compared with placebo in a RCT [78]. However, the effect was short lived, and no differences between groups were noted at 3 months.

## Future therapies

Given the success of rituximab, further work is ongoing to identify alternative therapies for B-cell depletion. A novel fully humanized monoclonal anti-CD20 antibody, ocrelizumab, has been trialed in rheumatoid arthritis [79]. Initial safety data suggest that ocrelizumab is well tolerated, rapidly depletes B cells with clinical improvement and has similar adverse effects when compared with standard treatment.

## Renal transplantation in AAV

Despite improved recognition and management of the AAV, progression to ESKD still occurs in a significant proportion of patients. While the paediatric literature is limited, both renal and non-renal relapses have been reported in adult patients with GPA, MPA and RLV. In a pooled analysis of published data, the recurrence rate for adults post-renal transplantation was reported to be 17.3% (127 patients), with no differences in relapse rate noted between the three disease entities. Average time from transplant to relapse was 31 months. Duration of time on dialysis, ANCA subtype, ANCA positivity at time of transplantation, allograft type (deceased or live) and immunosuppression regimen (with or without ciclosporin) had no clear effect on relapse rate [80].

More recently, a large retrospective study of 85 patients reported a low relapse rate of 0.02 per patient-year (median follow-up 64 months, range 3–165 months) on a prednisolone, MMF and tacrolimus regimen post-transplantation, with lower relapse rates than in patients on dialysis, perhaps the result of immunosuppression regimens used post-transplantation [81]. Renal function post-transplantation is also comparable to control subjects, with similar patient and graft survival rates seen at 1, 5 and 10 years of follow-up [81, 82]. Therefore, despite the perpetual risk of relapse, kidney transplantation is recommended for patients with ESKD during clinical remission, regardless of ANCA status [80, 81].

## Prognosis

Most of our knowledge regarding the long-term outcomes of AAV is based on adult data. In children, the majority of studies have a limited follow-up: to date, seven case series describe a total of 86 paediatric patients with AAV, with follow-up periods ranging from 4 months to 11 years. Compared with adult data, long-term disease or treatment-related morbidity is reportedly lower (22 vs. 45% [33]), however the data are weak and the burden is still significant. A recent study of children followed up into adulthood (median of 18.5 years, range 11–30 years [3]) described eight patients with relapsing disease. One death was noted secondary to respiratory complications of GPA. With regards to treatment-related morbidity, 50% of patients were infertile, 7 patients suffered infections, one patient developed a malignancy and two developed skeletal complications secondary to corticosteroid treatment. One-half of all patients developed ears, nose, and throat complications, including hearing loss (4 patients), nasal septum (2 patients) or upper airway deformities (1 patient). All but one patient had received cyclophosphamide, with five patients requiring repeated courses. Biological agents were used in three patients, with higher rates of infection seen with infliximab. The study emphasizes the need for effective immunosuppressive regimens while minimizing adverse effects: paediatric patients are at potentially greater risk of long-term morbidity due to their age and cumulative exposure and are therefore likely to benefit most from further research in this field.

## Conclusion

Anti-neutrophil cytoplasmic antibody-associated vasculitis is a rare condition in children, but one that is associated with significant morbidity and mortality. The exact pathogenesis remains unknown, and it is likely to be multifactorial. Understanding some of the immune mechanisms underlying the disease has led to the addition of biologic therapies as feasible management options. The evidence base for management, especially in children, is limited; however, early aggressive immunosuppression is strongly recommended to achieve a prompt disease remission, and subsequent careful monitoring is required to assess for disease relapse. European initiatives may provide standardized guidance on management in the near future. Renal involvement is often associated with a worse long-term outcome, although renal transplantation remains an option in those who progress to ESKD.
